# Hemodialysis versus peritoneal dialysis and diastolic blood pressure variability: volume-dependent cardiovascular risks in maintenance dialysis patients

**DOI:** 10.1080/0886022X.2026.2641980

**Published:** 2026-03-12

**Authors:** Yang Wu, Ting Yu, Dan Wu, Shan Ma, Yanqing Hu

**Affiliations:** ^a^ Department of Cardiology, The Seventh Affiliated Hospital, Sun Yat-sen University, Shenzhen, China; ^b^ Department of Endocrinology, The Seventh Affiliated Hospital, Sun Yat-sen University, Shenzhen, China; ^c^ Department of Emergency and Disaster Medical Center, The Seventh Affiliated Hospital, Sun Yat-sen University, Shenzhen, China; ^d^ Department of Orthopedics, The Seventh Affiliated Hospital, Sun Yat-sen University, Shenzhen, China

**Keywords:** Chronic kidney disease, maintenance dialysis, hemodialysis, peritoneal dialysis, diastolic blood pressure variability, volume management

## Abstract

This retrospective study analyzed hemodialysis (HD) and peritoneal dialysis (PD) impacts on diastolic blood pressure variability (DBPV) in Stage 5 chronic kidney disease (CKD-G5D) patients, explored volume-related indicator associations with DBPV, and provided clinical volume management evidence. Patients with CKD-G5D on maintenance dialysis (January 2019–December 2023) were included (*n* = 426; 298 HD, 128 PD), with median follow-up of 32.5 months. DBPV was assessed *via* 24-hour ambulatory blood pressure monitoring (ABPM), and volume indicators included interdialytic weight gain percentage (IDWG%), ultrafiltration volume (UFV), extracellular volume/body surface area (ECV/BSA), and N-terminal pro-B-type natriuretic peptide (NT-proBNP). HD patients had significantly higher 24h DBP SD (16.1 ± 5.3 vs 13.5 ± 4.5 mmHg), 24h DBP CV (21.3 ± 5.7% vs 17.9 ± 4.9%), and non-dipping DBPV prevalence compared with PD patients (70.1% vs 53.9%, all *p* < 0.01). IDWG% (*r* = 0.45) correlated strongest with 24h DBP SD, and dialysis modality (β = 2.31) and IDWG% (β = 1.95) were main DBPV influencers (both *p* < 0.001). 24h DBP SD ≥16 mmHg (dichotomous variable, HR = 1.15, 95% CI:1.08–1.23, *p* < 0.001) and HD modality independently predicted cardiovascular events. Targeted volume management improved DBPV in HD patients with IDWG% >5%. These results suggest that monitoring DBPV and IDWG% should be incorporated into routine dialysis care to mitigate cardiovascular risk.

## PLAIN LANGUAGE SUMMARY

In this study of 426 patients receiving long-term dialysis, those treated with hemodialysis showed greater daily fluctuations in diastolic blood pressure compared with peritoneal dialysis patients. These fluctuations were closely linked to fluid overload (measured by weight gain between dialysis sessions) and predicted heart and blood vessel complications. Careful fluid control (e.g., limiting salt/fluid intake, optimizing target weight) reduced blood pressure variability, suggesting a practical target for lowering heart and vascular risks in dialysis care.

## Graphical abstract

**Figure:**
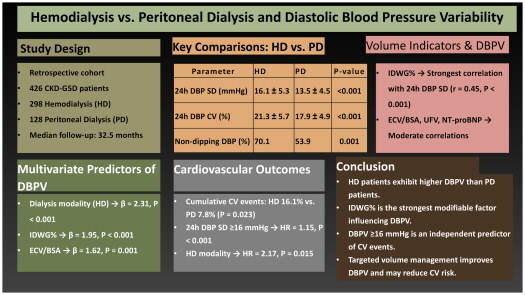

## 1. Introduction

Chronic kidney disease (CKD) has become a global public health challenge, with an estimated prevalence of over 10% among adults worldwide [1]. For patients with Stage 5 CKD (CKD-G5D), maintenance dialysis (including hemodialysis [HD] and peritoneal dialysis [PD]) is the primary renal replacement therapy to sustain life. However, despite advances in dialysis technology and adherence to the latest KDIGO guidelines for cardiovascular disease (CVD) management in CKD, cardiovascular disease (CVD) remains the leading cause of death in this population, with a mortality rate 10–20 times higher than that of the general population [2]. This excessive cardiovascular burden cannot be fully explained by traditional risk factors such as hypertension, diabetes, and dyslipidemia, highlighting the need to identify novel prognostic markers and intervention targets.

Diastolic blood pressure variability (DBPV), defined as short- or long-term fluctuations in diastolic blood pressure (DBP), has emerged as a critical indicator of cardiovascular health beyond static blood pressure values. Unlike systolic blood pressure variability (SBPV), which primarily reflects large-artery stiffness, DBPV is more closely associated with microvascular resistance, endothelial function, and cardiac relaxation [3]. Our recent studies have confirmed that elevated DBPV is an independent predictor of all-cause mortality, cardiovascular death, and heart failure hospitalization in CKD patients, especially in advanced stages [4]. However, the role of DBPV in CKD-G5D patients receiving maintenance dialysis remains understudied, and the impact of different dialysis modalities on DBPV has not been systematically evaluated.

HD and PD represent two distinct dialysis modalities with fundamental differences in volume regulation mechanisms. HD relies on intermittent ultrafiltration to remove excess fluid, leading to rapid fluctuations in intravascular volume between dialysis sessions (e.g., significant fluid accumulation during the interdialytic period and rapid fluid removal during dialysis) [5]. In contrast, PD achieves continuous fluid and solute clearance through peritoneal membrane exchange, resulting in more gradual and stable volume control [6]. These differences in volume dynamics may have profound effects on blood pressure stability, particularly DBPV. However, existing studies on this topic are limited by small sample sizes, short follow-up durations, and a focus on SBPV rather than DBPV. For example, a prospective study by Mallamaci et al. [7] included only 120 HD patients and found a correlation between volume overload and SBPV, but did not compare PD patients or evaluate DBPV. Another study by Churchill et al. [8] reported lower cardiovascular mortality in PD patients but did not explore the role of blood pressure variability.

Given these gaps, the present retrospective study aimed to address the following key questions: (1) Do HD and PD patients differ in DBPV parameters (24h DBP SD, 24h DBP CV, and non-dipping pattern)? (2) Which volume-related indicators (e.g., IDWG%, ECV/BSA) are associated with elevated DBPV in dialysis patients? (3) Is elevated DBPV an independent predictor of cardiovascular events in this population? (4) Can targeted volume management improve DBPV in high-risk patients (e.g., HD patients with high IDWG%)? By analyzing a large cohort of CKD-G5D patients with long-term follow-up, this study sought to provide evidence for optimizing volume management and reducing cardiovascular risks in maintenance dialysis patients. Notably, DBPV could serve as a physiological feature for future artificial intelligence models predicting cardiovascular events in dialysis populations, aligning with recent consensus on AI applications in nephrology [9].

## 2. Methods

### 2.1. Study design and participants

This retrospective cohort study was conducted at the Seventh Affiliated Hospital of Sun Yat-sen University, a tertiary referral center in Shenzhen, China. Patients with CKD-G5D undergoing maintenance dialysis were selected from the hospital’s EMR system and dialysis registry system between January 2019 and December 2023. The study protocol was approved by the Institutional Review Board in accordance with the Declaration of Helsinki.

Inclusion Criteria: (1) Age ≥18 and ≤80 years; (2) Diagnosis of CKD-G5D (estimated glomerular filtration rate [eGFR] < 15 mL/min/1.73m^2^) based on the KDIGO 2024 guidelines [10]; (3) Receiving maintenance HD for ≥3 months (3 sessions per week, 4 h per session) or maintenance PD for ≥3 months (continuous ambulatory peritoneal dialysis [CAPD] or automated peritoneal dialysis [APD]); (4) Completed at least one 24-h ABPM during dialysis treatment (HD patients on non-dialysis days) with an effective recording rate ≥80% (effective recording rate = number of valid readings/total scheduled readings × 100%, where valid readings were defined as systolic blood pressure [SBP] 70–250 mmHg, DBP 40–150 mmHg, and pulse rate 40–150 beats/min); (5) Complete medical records, including baseline demographic data, laboratory results, dialysis parameters, and follow-up information.

Exclusion Criteria: (1) Acute infections (e.g., pneumonia, urinary tract infection), acute coronary syndrome, or stroke within 3 months before ABPM; (2) History of malignant tumors (except for non-melanoma skin cancer); (3) Severe heart failure (New York Heart Association [NYHA] Class IV); (4) Severe liver dysfunction (Child-Pugh Class C); (5) Missing key data (e.g., IDWG%, ECV/BSA, or follow-up outcomes); (6) Pregnancy or lactation; (7) Irregular sleep schedules (e.g., shift workers) confounding ABPM circadian analysis.

**Figure 1.**
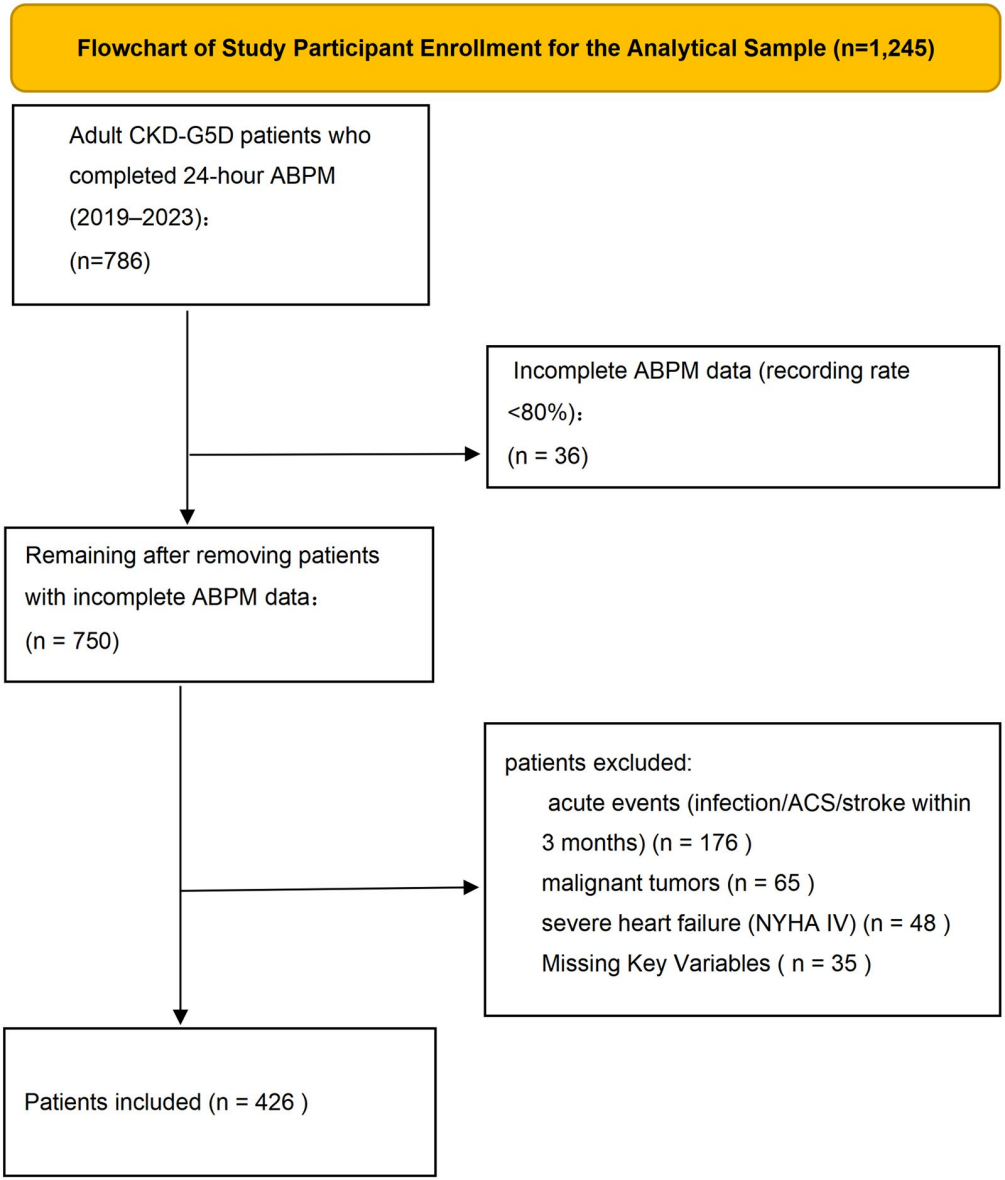
Patient selection flowchart (*n* = 426; 298 HD, 128 PD). A total of 786 CKD-G5D patients undergoing maintenance dialysis were initially screened. After applying exclusion criteria, 426 patients were included (298 HD, 128 PD). Created using Microsoft Excel 2021.

A flow diagram of patient selection is presented in Figure 1, which was created using Microsoft Excel 2021 (Microsoft Corporation, Redmond, WA, USA) to illustrate the stepwise exclusion process.

### 2.2. Data collection

Data were extracted from the hospital’s EMR system, dialysis registry system, and ABPM database by two independent researchers. Discrepancies were resolved through discussion with a senior nephrologist.

#### 2.2.1. Baseline data

Baseline data were collected at the time of the first ABPM (defined as the “baseline time point”). Demographic characteristics included age, sex, and body mass index (BMI, calculated as weight in kilograms divided by height in meters squared). Comorbidities included hypertension (defined as SBP ≥140 mmHg, DBP ≥90 mmHg, or use of antihypertensive medications), diabetes mellitus (defined as fasting blood glucose ≥7.0 mmol/L, 2-h postprandial blood glucose ≥11.1 mmol/L, glycated hemoglobin ≥6.5%, or use of antidiabetic medications), and coronary heart disease (confirmed by coronary angiography, myocardial infarction history, or typical angina symptoms with positive stress tests). Information on antihypertensive medication use (including renin-angiotensin-aldosterone system [RAAS] inhibitors, calcium channel blockers [CCBs], diuretics, and beta-blockers) was also recorded.

#### 2.2.2. DBPV monitoring

All patients underwent 24-h ABPM using a validated device (Omron HEM-7600CT, Omron Healthcare Co., Ltd., Kyoto, Japan) according to standardized protocols [11]. For HD patients, ABPM was performed on non-dialysis days to avoid acute hemodynamic changes during dialysis; PD patients had no specific timing restrictions. Patients were instructed to maintain their usual daily activities during monitoring, avoid strenuous exercise, alcohol consumption, and caffeine intake 2 h before measurement. Blood pressure readings were automatically obtained every 30 min during the daytime (06:00–22:00) and every 60 min during the nighttime (22:00–06:00). The following DBPV parameters were calculated: 24h DBP SD: The standard deviation of all valid 24-h DBP readings, reflecting absolute DBP variability; 24h DBP CV: Calculated as (24h DBP SD/24-h mean DBP) × 100%, reflecting relative DBP variability; Nocturnal DBP decline rate: Calculated as [(daytime mean DBP – nighttime mean DBP)/daytime mean DBP] × 100%. A decline rate <10% was defined as a “non-dipping DBP pattern,” which indicates abnormal circadian blood pressure regulation [12].

#### 2.2.3. Volume-Related indicators

Volume-related indicators were measured within 1 week after the baseline ABPM:

IDWG%: For HD patients, IDWG was defined as the weight gain between two consecutive dialysis sessions, and IDWG% was calculated as (IDWG/dry weight) × 100%. Dry weight was determined by experienced nephrologists based on clinical signs (e.g., absence of edema, normal jugular venous pressure) and bioelectrical impedance analysis (BIA). For PD patients, IDWG was defined as the weight difference before and after a complete PD exchange cycle, and IDWG% was calculated using the same formula to ensure comparability;

UFV: For HD patients, UFV was the total ultrafiltration volume during a single dialysis session, averaged over 1 month before baseline. For PD patients, UFV was the daily net ultrafiltration volume (total ultrafiltration volume minus dialysate inflow volume), averaged over 1 month before baseline;

ECV/BSA: ECV was measured using BIA (BCM, Fresenius), and BSA was calculated using the Du Bois formula [BSA (m^2^) = 0.007184 × height (cm)^0.725 × weight (kg)^0.425] for higher accuracy;

NT-proBNP: Serum NT-proBNP levels were measured using a chemiluminescent immunoassay (Roche Diagnostics, Mannheim, Germany), with a reference range <125 pg/mL for adults.

All HD sessions were standardized as high-flux hemodialysis without hemodiafiltration (HDF), with consistent dialysate composition and blood flow rate.

#### 2.2.4. Follow-Up and outcomes

The follow-up period began at the baseline ABPM time point and ended on December 31, 2023, or the first occurrence of the primary outcome. The primary outcome was a composite cardiovascular event, defined as the first occurrence of (1) heart failure hospitalization (confirmed by clinical symptoms, chest X-ray, and echocardiography), (2) acute myocardial infarction (confirmed by elevated cardiac troponin and typical electrocardiographic changes), or (3) cardiovascular death (death due to myocardial infarction, stroke, heart failure, or sudden cardiac arrest, as determined by death certificates or medical records). The secondary outcomes were defined as Ischemic stroke (confirmed by cranial CT/MRI), symptomatic cardiac arrhythmias (requiring antiarrhythmic medication or electrical cardioversion), and other vascular events (peripheral artery disease exacerbation, transient ischemic attack). Patients who were lost to follow-up (defined as no contact for >6 months) were censored at the last follow-up date. The cumulative incidence of cardiovascular events was visualized using stacked bar charts. To account for follow-up time heterogeneity, Cox proportional hazards regression was used to quantify event risk, with results presented alongside incidence data.

### 2.3. Statistical analysis

All statistical analyses were performed using SPSS 26.0 software (IBM Corporation, Armonk, NY, USA). The normality of continuous data was tested using the Shapiro-Wilk test. Normally distributed continuous data were presented as mean ± standard deviation (x ± s), and comparisons between groups were performed using the independent samples t-test. Non-normally distributed continuous data were presented as median (interquartile range [IQR]), and comparisons were performed using the Mann-Whitney U test. Categorical data were presented as counts (percentages), and comparisons between groups were performed using the chi-square test or Fisher’s exact test (if the expected frequency was <5).

Correlations between volume-related indicators and 24h DBP SD were analyzed using Pearson correlation coefficients (for normally distributed data) or Spearman rank correlation coefficients (for non-normally distributed data). The strength of correlations was interpreted as follows: |r| <0.1 (no correlation), 0.1–0.3 (weak correlation), 0.3–0.5 (moderate correlation), and >0.5 (strong correlation) [13].

Multivariate linear regression analysis was performed to identify factors influencing 24h DBP SD. Variables with *p* < 0.1 in univariate analysis (including dialysis modality, IDWG%, ECV/BSA, NT-proBNP, age, sex, BMI, hypertension, diabetes, and coronary heart disease) were included in the multivariate model. Collinearity between variables was assessed using the variance inflation factor (VIF), with VIF <5 indicating no significant collinearity [14].

The association between DBPV and cardiovascular events was primarily analyzed using Cox proportional hazards regression. Cumulative incidence was presented for descriptive purposes, with statistical significance verified by Cox models. The proportional hazards assumption was verified using Schoenfeld residuals. Univariate Cox regression was first performed to screen variables associated with cardiovascular events (*p* < 0.1). Multivariate Cox regression was then performed to adjust for potential confounders, including age, sex, BMI, diabetes, coronary heart disease, and hypertension. Hazard ratios (HRs) and 95% CIs were reported. To clarify the HR value, 24h DBP SD was analyzed as both a dichotomous variable (≥16 mmHg vs <16 mmHg) and a continuous variable (per 1 mmHg increase).

For the subgroup analysis of volume management, paired t-tests (for continuous data) and McNemar’s test (for categorical data) were used to compare DBPV parameters before and after intervention in HD patients with IDWG% >5%.

A two-sided *p* < 0.05 was considered statistically significant for all analyses. Graphs (including the patient flow diagram, bar charts, scatter plots, forest plots, and stacked bar charts for cumulative incidence) were created using Microsoft Excel 2021, with error bars representing 95% CIs for continuous variables.

## 3. Results

### 3.1. Patient selection and baseline characteristics

A total of 786 CKD-G5D patients undergoing maintenance dialysis were initially screened from the EMR system and dialysis registry system. After applying the exclusion criteria (30 patients with invalid ABPM, 182 patients with acute events within 3 months, 65 patients with malignant tumors, 48 patients with severe heart failure, 35 patients with missing key data, and 4 with irregular sleep schedules), 426 patients were finally included in the study (Figure 1, *n* = 426; 298 HD, 128 PD).

The baseline characteristics of the two groups are presented in Table 1 (*n* = 426; 298 HD, 128 PD). There were no significant differences in age, sex, BMI, or the prevalence of comorbidities (hypertension, diabetes, coronary heart disease) between the HD and PD groups (all *p* > 0.05). However, volume-related indicators differed significantly between the two groups: the HD group had higher IDWG% (5.1 ± 1.6% vs 3.3 ± 1.2%, *p* < 0.001), higher UFV (2.9 ± 0.9 L vs 1.6 ± 0.7 L, *p* < 0.001), higher ECV/BSA (9.4 ± 1.4 L/m^2^ vs 8.2 ± 1.1 L/m^2^, *p* < 0.001), and higher NT-proBNP levels (1620 [850–2410] pg/mL vs 980 [530–1650] pg/mL, *p* < 0.001) than the PD group. The use of antihypertensive medications (RAAS inhibitors, CCBs, diuretics, beta-blockers) was similar between the two groups (all *p* > 0.05).

**Table 1.** Baseline characteristics of the HD and PD groups (*n* = 426; 298 HD, 128 PD).

Variable	HD group (*n* = 298)	PD group (*n* = 128)	Test statistic	*p* Value
Demographics				
Age (years), mean ± SD	64.2 ± 11.5	63.5 ± 10.8	*t* = 0.57	0.568
Male sex, *n* (%)	168 (56.4)	72 (56.3)	χ²=0.001	0.972
BMI (kg/m²), mean ± SD	25.1 ± 3.8	24.7 ± 3.6	*t* = 0.92	0.358
Comorbidities, *n* (%)				
Hypertension	272 (91.3)	117 (91.4)	χ²=0.002	0.964
Diabetes mellitus	121 (40.6)	52 (40.6)	χ²=0.000	1.000
Coronary heart disease	68 (22.8)	27 (21.1)	χ²=0.22	0.639
Volume-related indicators				
IDWG%, mean ± SD	5.1 ± 1.6	3.3 ± 1.2	*t* = 11.24	<0.001
UFV (L), mean ± SD	2.9 ± 0.9	1.6 ± 0.7	*t* = 16.87	<0.001
ECV/BSA (L/m²), mean ± SD	9.4 ± 1.4	8.2 ± 1.1	*t* = 8.92	<0.001
NT-proBNP (pg/mL), M (IQR)	1620 (850–2410)	980 (530–1650)	*Z* = 6.15	<0.001
Antihypertensive medications, n (%)				
RAAS inhibitors	156 (52.3)	67 (52.3)	χ²=0.000	1.000
Calcium channel blockers	182 (61.1)	78 (61.0)	χ²=0.001	0.974
Diuretics	75 (25.2)	32 (25.0)	χ²=0.002	0.963
Beta-blockers	98 (32.9)	42 (32.8)	χ²=0.001	0.973

Abbreviations: HD: Hemodialysis; PD: Peritoneal Dialysis; BMI: Body Mass Index; IDWG%: Interdialytic Weight Gain Percentage; UFV: Ultrafiltration Volume; ECV/BSA: Extracellular Volume/Body Surface Area; NT-proBNP: N-terminal Pro-B-type Natriuretic Peptide; RAAS: Renin-Angiotensin-Aldosterone System; M (IQR): Median (Interquartile Range).

For the volume management subgroup, 92 HD patients (30.9%) had IDWG% >5%. Baseline characteristics between the intervention group (*n* = 65) and nonintervention group (*n* = 27) were balanced: 24h DBP SD (17.5 ± 5.1 vs 17.8 ± 4.9 mmHg, *p* = 0.76), IDWG% (6.2 ± 1.3% vs 6.3 ± 1.2%, *p* = 0.81), ECV/BSA (9.7 ± 1.5 vs 9.8 ± 1.4 L/m^2^, *p* = 0.73), and comorbidities (all *p* > 0.05).

### 3.2. Comparison of DBPV parameters between the HD and PD groups

**Figure 2.**
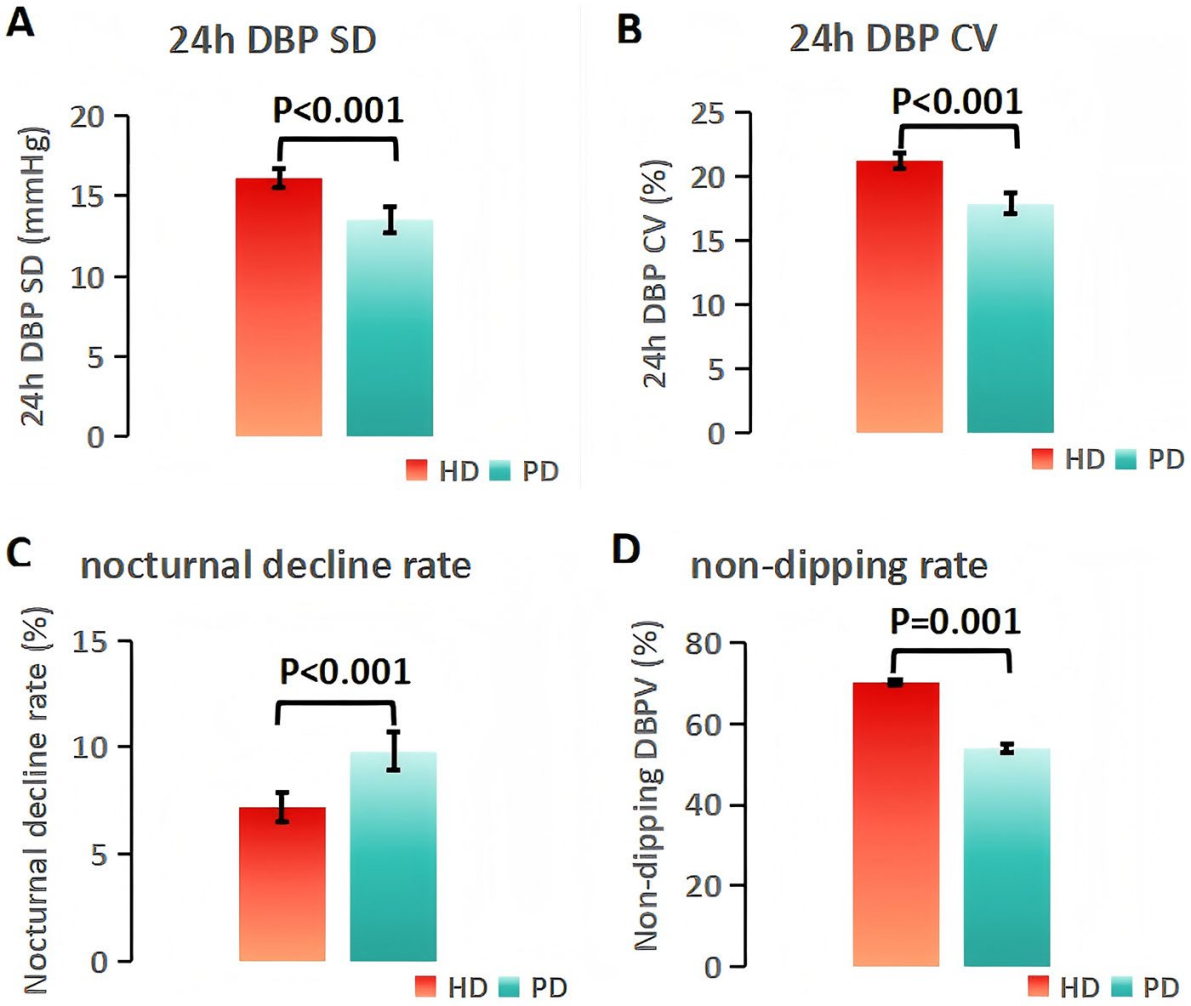
Comparison of DBPV Parameters (*n* = 426; 298 HD, 128 PD). (A) 24h DBP SD, (B) 24h DBP CV, (C) nocturnal DBP decline rate, (D) non-dipping prevalence. HD patients had significantly higher DBPV. Error bars: 95% CI. Abbreviations: DBPV = diastolic blood pressure variability; HD = hemodialysis; PD = peritoneal dialysis.

The DBPV parameters of the two groups are presented in Figure 2 (*n* = 426; 298 HD, 128 PD). The HD group had significantly higher 24h DBP SD (16.1 ± 5.3 mmHg vs 13.5 ± 4.5 mmHg, *p* < 0.001) and 24h DBP CV (21.3 ± 5.7% vs 17.9 ± 4.9%, *p* < 0.001) than the PD group. The prevalence of non-dipping DBPV was also significantly higher in the HD group (70.1% vs 53.9%, *p* = 0.001). In addition, the HD group had a lower nocturnal DBP decline rate (7.2 ± 6.8% vs 9.8 ± 6.1%, *p* < 0.001), indicating more severe circadian blood pressure dysregulation.

### 3.3. Correlation between volume-related indicators and DBPV

**Figure 3.**
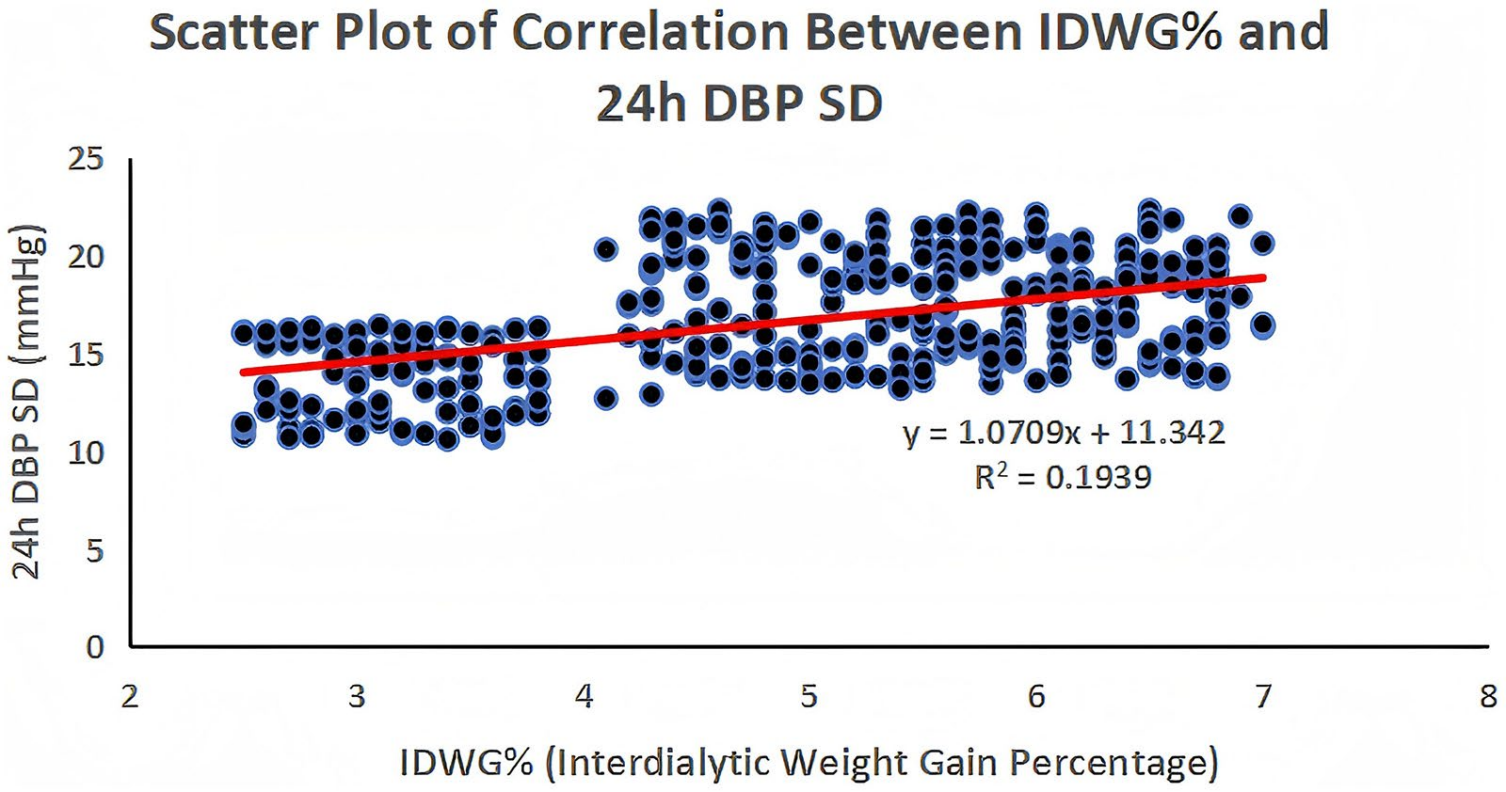
Correlation Between IDWG% and 24h DBP SD (*n* = 426; 298 HD, 128 PD). Each dot = patient; red line = linear regression. IDWG% showed moderate positive correlation (r = 0.45, P < 0.001). Abbreviations: IDWG%=interdialytic weight gain percentage; 24h DBP SD = 24-hour diastolic blood pressure standard deviation.

The correlations between volume-related indicators and 24h DBP SD are presented in Supplementary Table S1 and Figure 3 (*n* = 426; 298 HD, 128 PD). IDWG% showed the strongest positive correlation with 24h DBP SD (*r* = 0.45, *p* < 0.001), followed by ECV/BSA (*r* = 0.38, *p* < 0.001), UFV (*r* = 0.36, *p* < 0.001), and NT-proBNP (*r* = 0.33, *p* < 0.001). All correlations were moderate in strength, indicating that higher volume overload was associated with greater DBP variability.

### 3.4. Factors Influencing DBPV: Multivariate linear regression analysis

Univariate linear regression analysis showed that dialysis modality (HD vs PD), IDWG%, ECV/BSA, NT-proBNP, age, and diabetes were associated with 24h DBP SD (all *p* < 0.1). These variables were included in the multivariate linear regression model (VIF <3 for all variables, indicating no collinearity).

**Figure 4.**
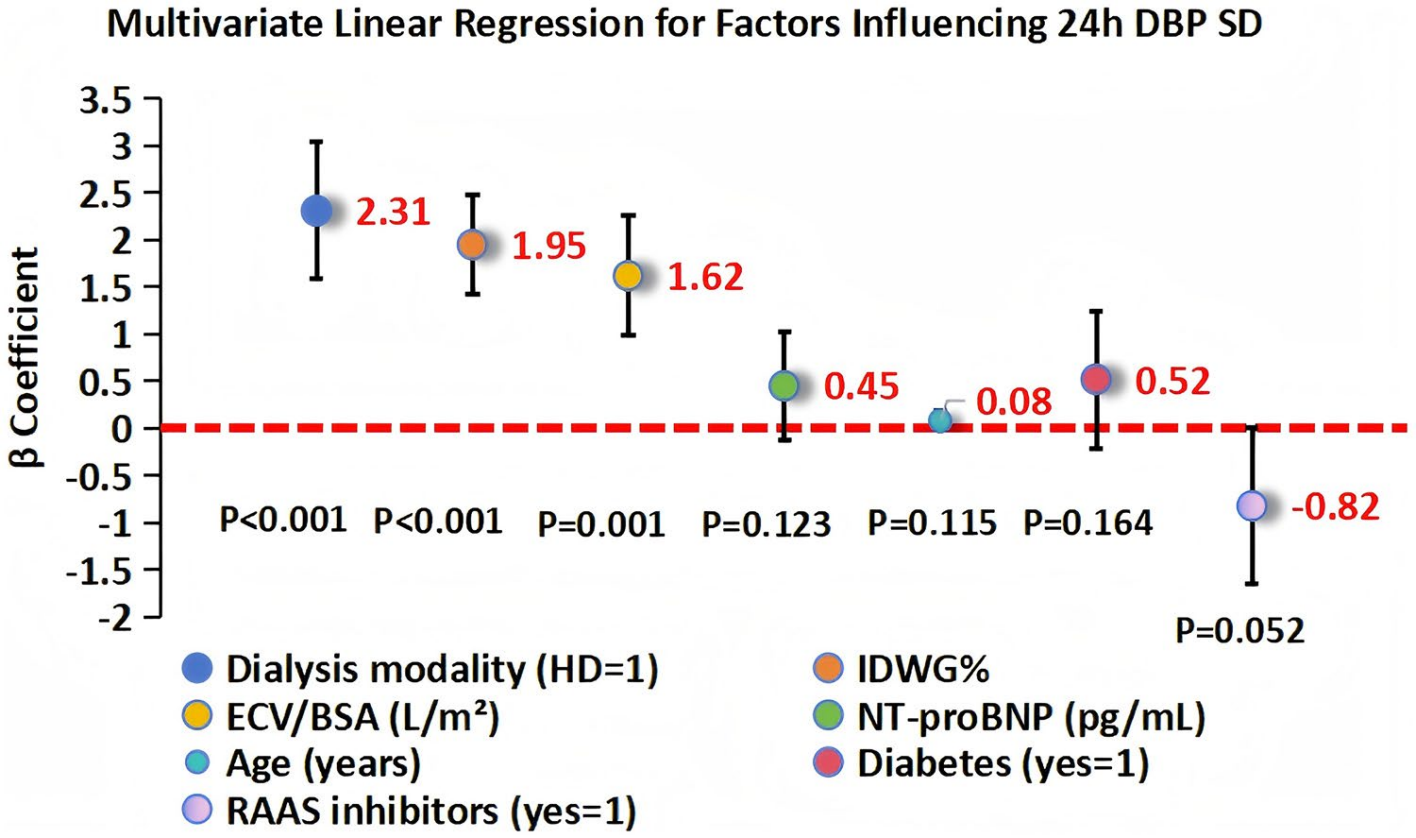
Forest plot of factors influencing 24h DBP SD (*n* = 426; 298 HD, 128 PD). Dialysis modality, IDWG%, and ECV/BSA were independent predictors. β = beta coefficient; CI = confidence interval.

The results of the multivariate linear regression are presented in Table 2 and Figure 4 (*n* = 426; 298 HD, 128 PD). Dialysis modality (β = 2.31, 95% CI: 1.58–3.04, *p* < 0.001) and IDWG% (β = 1.95, 95% CI: 1.42–2.48, *p* < 0.001) were the most significant predictors of 24h DBP SD. ECV/BSA also independently predicted 24h DBP SD (β = 1.62, 95% CI: 0.98–2.26, *p* = 0.001), while NT-proBNP, age, diabetes and RAAS inhibitors were not significant after adjustment (all *p* > 0.05). The model explained 41% of the variance in 24h DBP SD (R^2^=0.41, *F* = 32.75, *p* < 0.001).

**Table 2.** Multivariate linear regression analysis of factors Influencing 24h DBP SD.

Variable	β Coefficient	95% CI	*p* Value
Dialysis modality (HD = 1, PD = 0)	2.31	1.58–3.04	<0.001
IDWG%	1.95	1.42–2.48	<0.001
ECV/BSA (L/m²)	1.62	0.98–2.26	0.001
NT-proBNP (pg/mL)	0.45	−0.12–1.02	0.123
Age (years)	0.08	−0.02–0.18	0.115
Diabetes (yes = 1, no = 0)	0.52	−0.21–1.25	0.164
RAAS inhibitors (yes = 1)	−0.82	−1.65–0.01	0.052

Abbreviations: 24h DBP SD: 24-h DBP Standard Deviation; HD: Hemodialysis; PD: Peritoneal Dialysis; IDWG%: Interdialytic Weight Gain Percentage; ECV/BSA: Extracellular Volume/Body Surface Area; NT-proBNP: N-terminal Pro-B-type Natriuretic Peptide; CI: Confidence Interval.

### 3.5. Follow-Up Outcomes and cardiovascular risk analysis

**Figure 5.**
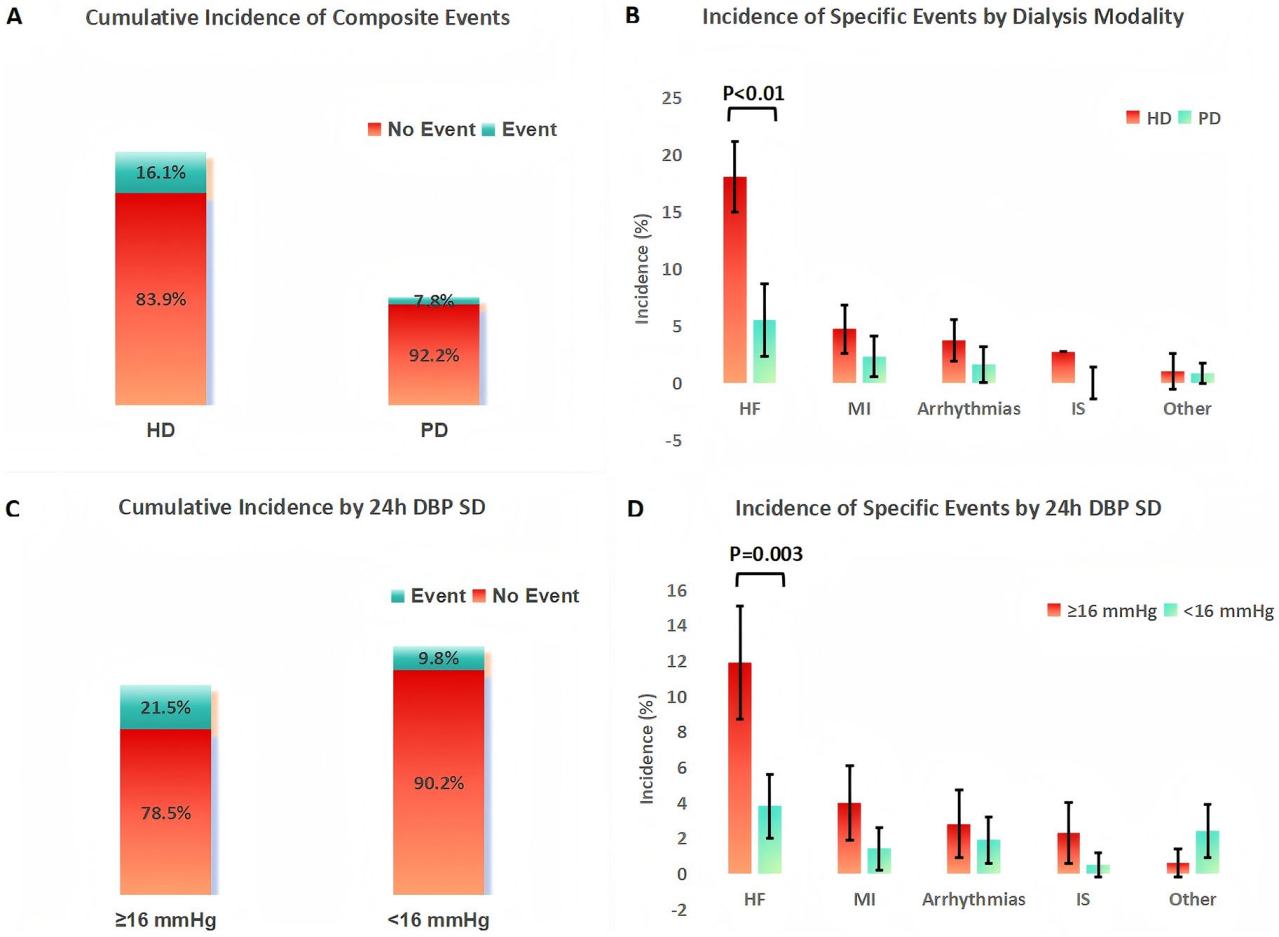
Cumulative incidence of cardiovascular events (*n* = 426; 298 HD, 128 PD). (A) By dialysis modality (HD:16.1% vs PD:7.8%, P = 0.023), (B) by 24h DBP SD (≥16 mmHg:21.5% vs <16 mmHg:9.8%, P < 0.001). Error bars:95% CI.

The median follow-up duration was 32.5 months (IQR: 20.1–45.3 months), during which 58 primary composite cardiovascular events were documented, with findings visualized in Figure 5 (*n* = 426; 298 HD, 128 PD). The cumulative incidence of cardiovascular events in the HD group was higher than in the PD group (16.1% vs. 7.8%, χ^2^=5.18, *p* = 0.023). Cox regression confirmed this association after adjusting for follow-up duration (HR = 2.03, 95% CI: 1.08–3.82, *p* = 0.029).

When categorized by primary event type, heart failure (HF) hospitalization emerged as the most common complication, accounting for 43.1% (25/58) of all primary events, followed by acute myocardial infarction (MI, 24.1%, 14/58) and cardiovascular death (32.8%, 19/58). Among all HF-related events (a secondary outcome, including HF hospitalization and clinically confirmed outpatient HF exacerbation), 29 occurred in HD patients (9.7%, 29/298) and 7 in PD patients (5.5%, 7/128), representing the most frequent adverse cardiovascular outcome in both groups but with a significantly higher incidence in HD (χ^2^=8.76, *p* = 0.003). A total of 31 secondary cardiovascular events were recorded during follow-up, including arrhythmias (17.2%, 9/53) and ischemic stroke (10.3%, 5/53) (excluding primary events). After adjusting for age, diabetes, and baseline ECV/BSA, HD remained independently associated with a higher risk of composite primary cardiovascular events (adjusted HR = 2.17, 95% CI: 1.16–4.06, *p* = 0.015), with HF hospitalization driving this association (adjusted HR = 3.24, 95% CI: 1.21–8.69, *p* = 0.020).

When stratified by 24h DBP SD, patients with ≥16 mmHg had a higher cumulative incidence (21.5% vs. 9.8%, χ^2^=12.36, *p* < 0.001), with Cox regression showing an adjusted HR = 2.53 (95% CI: 1.46–4.39, *p* = 0.001) (stratified analysis). As a dichotomous variable, 24h DBP SD ≥16 mmHg independently predicted cardiovascular events (HR = 1.15, 95% CI: 1.08–1.23, *p* < 0.001) in multivariate Cox regression. When analyzed as a continuous variable, each 1 mmHg increase in 24h DBP SD was independently associated with a 5% higher risk of cardiovascular events (HR = 1.05, 95% CI: 1.02–1.08, *p* < 0.001), confirming a dose-response relationship.

**Figure 6.**
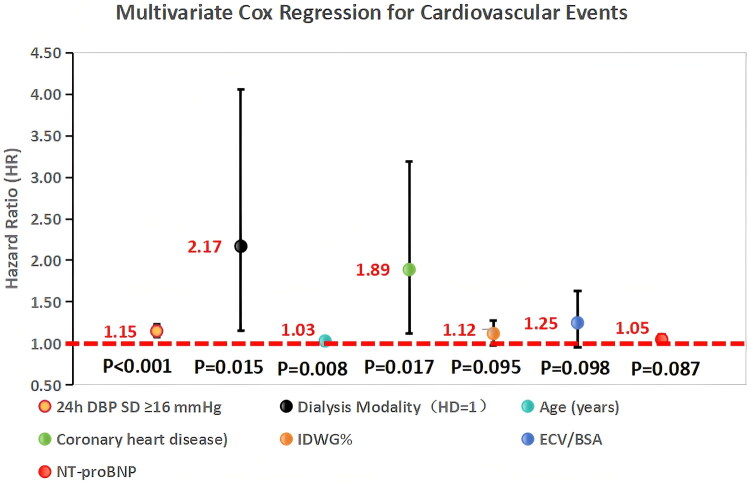
Forest plot of cardiovascular event predictors (*n* = 426; 298 HD, 128 PD). 24h DBP SD ≥16 mmHg, HD modality, age, and coronary heart disease were independent predictors. HR = hazard ratio; CI = confidence interval.

The results of the multivariate Cox regression are presented in Table 3 and Figure 6 (*n* = 426; 298 HD, 128 PD). 24h DBP SD ≥16 mmHg (HR = 1.15), HD modality (HR = 2.17), age (HR = 1.03), and coronary heart disease (HR = 1.89) were independent predictors of cardiovascular events (all *p* < 0.05). IDWG%, ECV/BSA, and NT-proBNP were not significant after adjustment (all *p* > 0.05).

**Table 3.** Multivariate Cox regression analysis of factors Influencing cardiovascular events.

Variable	HR	95% CI	*p* Value
24h DBP SD ≥16 mmHg (yes = 1, no = 0)	1.15	1.08–1.23	<0.001
Dialysis modality (HD = 1, PD = 0)	2.17	1.16–4.06	0.015
Age (years)	1.03	1.01–1.05	0.008
Coronary heart disease (yes = 1, no = 0)	1.89	1.12–3.19	0.017
IDWG%	1.12	0.98–1.28	0.095
ECV/BSA (L/m²)	1.25	0.96–1.63	0.098
NT-proBNP (pg/mL)	1.05	0.99–1.11	0.087

Abbreviations: HR: Hazard Ratio; CI: Confidence Interval; 24h DBP SD: 24-h DBP Standard Deviation; HD: Hemodialysis; PD: Peritoneal Dialysis; IDWG%: Interdialytic Weight Gain Percentage; ECV/BSA: Extracellular Volume/Body Surface Area; NT-proBNP: N-terminal Pro-B-type Natriuretic Peptide.

### 3.6. Subgroup analysis of volume management in HD patients

Targeted volume management included: (1) Dietary counseling to reduce salt intake (<5 g/day) and fluid intake (<1000 mL/day); (2) Optimization of dry weight using BIA (adjusted by 0.5–1.0 kg based on ECV/BSA); (3) Increased HD frequency (from 3 to 4 sessions per week) or prolonged HD duration (from 4 to 5 h per session) for patients with persistent volume overload.

**Figure 7.**
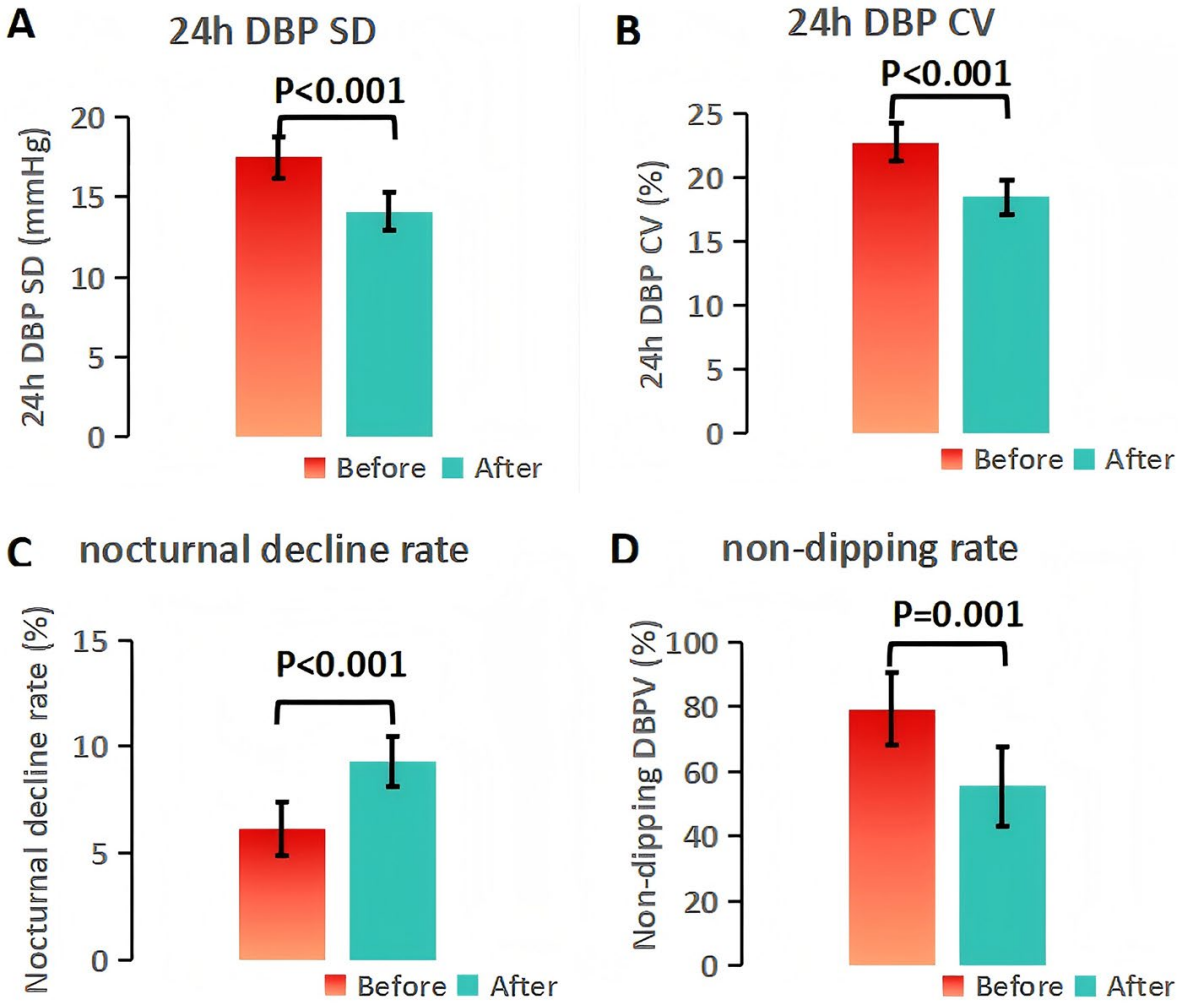
Impact of volume management in HD patients with IDWG% >5% (*n* = 65). (A) 24h DBP SD, (B) 24h DBP CV, (C) nocturnal decline rate, (D) non-dipping prevalence improved significantly after 3 months. Error bars:95% CI.

The DBPV parameters before and after volume management are presented in Figure 7 (*n* = 65). After 3 months of intervention, 24h DBP SD significantly decreased from 17.5 ± 5.1 to 14.1 ± 4.7 mmHg (*t* = 6.28, *p* < 0.001), and 24h DBP CV significantly decreased from 22.8 ± 5.9% to 18.5 ± 4.8% (*t* = 5.73, *p* < 0.001). The nocturnal DBP decline rate significantly increased from 6.1 ± 6.5% to 9.3 ± 5.8% (*t* = 3.87, *p* < 0.001), and the prevalence of non-dipping DBPV significantly decreased from 79.4% to 55.4% (χ^2^=10.15, *p* = 0.001). No significant adverse events (e.g., hypotension, muscle cramps) were reported during the intervention.

## 4. Discussion

This retrospective study of 426 CKD-G5D patients with a median follow-up of 32.5 months provides three key findings: (1) HD patients have significantly higher DBPV than PD patients, as evidenced by higher 24h DBP SD, 24h DBP CV, and a higher prevalence of non-dipping DBPV; (2) IDWG% is the strongest modifiable factor influencing DBPV, with each 1% increase in IDWG associated with a 1.95 mmHg increase in 24h DBP SD; (3) Targeted volume management improves DBPV in HD patients with high IDWG%, which may contribute to lower cardiovascular risks. These findings address critical gaps in the literature and have important implications for clinical practice.

### 4.1. Mechanisms underlying higher DBPV in HD patients

The observed difference in DBPV between HD and PD patients can be attributed to fundamental differences in their volume regulation mechanisms. HD relies on intermittent ultrafiltration, which creates a “volume swing” between dialysis sessions: during the interdialytic period, patients often accumulate 2–4 L of fluid (reflected by high IDWG%), leading to increased intravascular volume and elevated blood pressure [15]. During dialysis, this fluid is rapidly removed (often 2–3 L per session), causing a sudden decrease in intravascular volume and a subsequent drop in blood pressure. This rapid “rise and fall” in volume disrupts the stability of the cardiovascular system, activating the sympathetic nervous system and RAAS [16]. Sympathetic activation increases peripheral vascular resistance, while RAAS activation promotes sodium and water retention, both of which exacerbate DBP fluctuations [17]. In contrast, PD achieves continuous fluid removal through the peritoneal membrane, with a daily net ultrafiltration volume of only 1–2 L. This gradual volume control avoids large intravascular volume changes, maintaining more stable blood pressure and lower DBPV [18].

Another potential mechanism is the impact of dialysis modality on endothelial function. HD patients often experience repeated episodes of ischemia-reperfusion injury during dialysis, which impairs endothelial nitric oxide (NO) production [19]. NO is a key vasodilator that helps buffer blood pressure fluctuations; reduced NO levels increase vascular stiffness and decrease the ability of blood vessels to adapt to volume changes, thereby increasing DBPV [20]. PD patients, however, avoid ischemia-reperfusion injury, and the peritoneal membrane provides a more biocompatible environment, preserving endothelial function and reducing DBPV [21].

### 4.2. The Central role of IDWG in regulating DBPV

Our study confirms that IDWG% is the strongest modifiable factor influencing DBPV, with a moderate positive correlation (*r* = 0.45) and an independent predictive effect in multivariate analysis (β = 1.95). This finding aligns with the Dialysis Outcomes and Practice Patterns Study (DOPPS), which identified IDWG% >5% as a risk factor for cardiovascular mortality in HD patients [22]. High IDWG% reflects poor interdialytic volume control, leading to two key consequences that increase DBPV: (1) Interdialytic volume overload increases cardiac preload and afterload, elevating DBP and creating a higher “baseline” for fluctuations; (2) Rapid ultrafiltration during dialysis causes intravascular volume depletion, triggering reflex sympathetic activation and vasoconstriction, which further amplifies DBP swings [15].

The clinical significance of IDWG% as a therapeutic target is underscored by our subgroup analysis, which showed that targeted volume management—including dietary salt and fluid restriction, BIA-guided dry weight optimization, and adjusted dialysis prescriptions—significantly reduces DBPV in HD patients with IDWG% >5%. These interventions address the root cause of elevated DBPV in HD patients: volume overload and acute volume fluctuations. Importantly, the intervention was well-tolerated, with no significant adverse events, highlighting its feasibility in clinical practice.

### 4.3. Clinical significance of DBPV as a cardiovascular risk marker

Our study demonstrates that elevated DBPV (24h DBP SD ≥16 mmHg) is an independent predictor of cardiovascular events in maintenance dialysis patients, with an adjusted HR of 1.15 for the dichotomous variable and a 5% increase in risk per 1 mmHg increase in 24h DBP SD (continuous variable). This prognostic value is independent of traditional risk factors such as age, coronary heart disease, and dialysis modality, highlighting DBPV as a novel, quantifiable risk marker. The association between DBPV and cardiovascular events is particularly strong for heart failure, which accounted for over half of events in the high-variability group. This specificity is biologically plausible, as DBPV reflects microvascular resistance and cardiac relaxation—pathways that are central to the development of heart failure in CKD [4].

The mechanisms by which elevated DBPV increases cardiovascular risk include: (1) Increased mechanical stress on blood vessel walls, accelerating atherosclerosis and endothelial damage [23]; (2) Disruption of the circadian blood pressure rhythm (non-dipping pattern), which increases myocardial oxygen consumption during sleep and promotes cardiac remodeling [24]; (3) Exacerbation of microvascular damage, leading to organ hypoperfusion and dysfunction [25]. These mechanisms are particularly relevant in dialysis patients, who already have a high burden of cardiovascular risk factors and structural heart disease.

Our findings extend the results of landmark studies such as the Chronic Renal Insufficiency Cohort (CRIC) and the African American Study of Kidney Disease and Hypertension (AASK). The CRIC study demonstrated that volume overload is associated with higher BPV and increased cardiovascular risk in CKD patients [12], while AASK confirmed that BPV predicts cardiovascular outcomes in CKD [13]. By focusing on DBPV and demonstrating its prognostic value in the dialysis population, our study adds to the growing body of evidence supporting BPV as a critical cardiovascular risk marker in CKD.

### 4.4. Strengths and limitations

The strengths of our study include: (1) A large sample size (426 patients) and long median follow-up (32.5 months), providing adequate statistical power to detect differences in DBPV and its association with cardiovascular events; (2) Use of gold-standard 24-h ABPM to assess DBPV, with measurements timed on non-dialysis days for HD patients to avoid acute dialysis-related hemodynamic perturbations; (3) Comprehensive assessment of volume-related indicators, including IDWG%, ECV/BSA, and NT-proBNP, allowing for detailed analysis of determinants of DBPV; (4) A subgroup analysis of volume management, demonstrating the feasibility and efficacy of targeted interventions to reduce DBPV.

Despite these strengths, our study has several limitations that should be acknowledged: 1) Retrospective design and selection bias: Only patients with complete medical records and valid ABPM data were included, which may exclude patients with severe comorbidities or poor follow-up compliance. 2) Single-center setting: The study was conducted at a single tertiary hospital, limiting the generalizability of results to other populations and healthcare settings. 3) Non-randomized subgroup analysis of volume management: Patients who received intervention may have had better compliance with medical recommendations, which could confound the association between volume management and DBPV improvement. 4) Lack of longitudinal DBPV data: Only baseline DBPV was measured, and dynamic changes in DBPV during follow-up were not assessed. 5) Unmeasured confounding factors: Several potential confounders were not evaluated, including vascular stiffness (e.g., pulse wave velocity), oxidative stress markers (e.g., malondialdehyde), and sympathetic nervous system activity (e.g., heart rate variability). 6) Heterogeneity of volume management interventions: The relative contributions of individual components (e.g., salt restriction vs. increased dialysis frequency) to DBPV improvement could not be disentangled.

### 4.5. Future research directions

Based on our findings, future research should focus on: (1) Conducting multicenter prospective studies to validate the prognostic value of DBPV in diverse dialysis populations; (2) Exploring the mechanisms underlying the association between IDWG and DBPV, such as the role of endothelial function and sympathetic activation; (3) Evaluating the efficacy of DBPV-guided volume management in randomized controlled trials (e.g., comparing DBPV-guided vs standard volume management); (4) Develop AI-driven predictive models integrating DBPV with clinical, biochemical, and dialysis-related data, building on established frameworks and consensus statements for AI in kidney disease research and clinical practic [9,26]; (5) Developing noninvasive, real-time DBPV monitoring tools (e.g., wearable devices) to facilitate long-term DBPV management in dialysis patients; (6) Investigating whether reducing DBPV through targeted interventions translates to lower cardiovascular mortality in HD patients [27].

## 5. Conclusion

In conclusion, this retrospective study demonstrates that HD patients with CKD-G5D have significantly higher DBPV than PD patients, with IDWG% being the key modifiable factor. Elevated DBPV is an independent predictor of cardiovascular events, particularly heart failure, in maintenance dialysis patients. Targeted volume management improves DBPV in high-risk HD patients, and DBPV monitoring combined with IDWG% assessment should be integrated into routine dialysis care to reduce cardiovascular risk.

### 5.1. Clinical perspective

This study identifies DBPV ≥16 mmHg as a novel, quantifiable marker of volume-dependent cardiovascular risk among dialysis patients. Integration of ABPM metrics (e.g., 24h DBP SD) into routine volume assessment could guide personalized dialysis prescriptions (e.g., optimized dry weight, increased dialysis frequency). For HD patients with IDWG% >5%, targeted volume management (salt/fluid restriction, BIA-guided dry weight adjustment) is a practical strategy to improve hemodynamic stability and mitigate heart failure risk. By prioritizing DBPV monitoring and volume control, clinicians can adopt a more personalized approach to dialysis care, addressing a key modifiable driver of cardiovascular mortality in this high-risk population.

## Supplementary Material

Supplemental Material

## Data Availability

The datasets generated and analyzed during the current study are available from the corresponding authors (Y.W. and Y.H.) upon reasonable request. To ensure data integrity and patient privacy, a formal data sharing agreement may be required, which will comply with all relevant data protection regulations. The materials used in the study, including ABPM protocols and volume management guidelines, are also available from the corresponding authors upon request.
