# Supplementary material for: Hemodialysis versus peritoneal dialysis and diastolic blood pressure variability: volume-dependent cardiovascular risks in maintenance dialysis patients
Source: Ren Fail. 2026 Mar 12;48(1):2641980. doi: 10.1080/0886022X.2026.2641980 (PMC12983800; doi:10.1080/0886022X.2026.2641980)
Supplement: Supplemental Material [file IRNF_A_2641980_SM1978.docx]

**Supplementary Table S1: Correlation Between Volume-Related Indicators and 24h DBP SD**

| **Volume-Related Indicator** | **Correlation Coefficient**  **(r)** | **P Value** | **Correlation Strength** |
| --- | --- | --- | --- |
| IDWG% | 0.45 | <0.001 | Moderate |
| UFV (L) | 0.36 | <0.001 | Moderate |
| ECV/BSA (L/m²) | 0.38 | <0.001 | Moderate |
| NT-proBNP (pg/mL) | 0.33 | <0.001 | Moderate |

Abbreviations: 24h DBP SD=24-hour DBP Standard Deviation; IDWG%=Interdialytic Weight Gain Percentage; UFV=Ultrafiltration Volume; ECV/BSA=Extracellular Volume/Body Surface Area; NT-proBNP=N-terminal Pro-B-type Natriuretic Peptide.
